# Extracapsular extension risk assessment using an artificial intelligence prostate cancer mapping algorithm

**DOI:** 10.1002/bco2.421

**Published:** 2024-08-26

**Authors:** Alan Priester, Sakina Mohammed Mota, Kyla P. Grunden, Joshua Shubert, Shannon Richardson, Anthony Sisk, Ely R. Felker, James Sayre, Leonard S. Marks, Shyam Natarajan, Wayne G. Brisbane

**Affiliations:** ^1^ Avenda Health, Inc. United States; ^2^ Department of Urology David Geffen School of Medicine United States; ^3^ Department of Pathology David Geffen School of Medicine United States; ^4^ Department of Radiology David Geffen School of Medicine United States; ^5^ Department of Radiological Sciences and Biostatistics University of California, Los Angeles United States

**Keywords:** artificial intelligence, fusion biopsy, extracapsular extension, MRI, prostate cancer

## Abstract

**Objective:**

The objective of this study is to compare detection rates of extracapsular extension (ECE) of prostate cancer (PCa) using artificial intelligence (AI)‐generated cancer maps versus MRI and conventional nomograms.

**Materials and methods:**

We retrospectively analysed data from 147 patients who received MRI‐targeted biopsy and subsequent radical prostatectomy between September 2016 and May 2022. AI‐based software cleared by the United States Food and Drug Administration (Unfold AI, Avenda Health) was used to map 3D cancer probability and estimate ECE risk. Conventional ECE predictors including MRI Likert scores, capsular contact length of MRI‐visible lesions, PSMA T stage, Partin tables, and the “PRedicting ExtraCapsular Extension” nomogram were used for comparison.

Postsurgical specimens were processed using whole‐mount histopathology sectioning, and a genitourinary pathologist assessed each quadrant for ECE presence. ECE predictors were then evaluated on the patient (Unfold AI versus all comparators) and quadrant level (Unfold AI versus MRI Likert score). Receiver operator characteristic curves were generated and compared using DeLong's test.

**Results:**

Unfold AI had a significantly higher area under the curve (AUC = 0.81) than other predictors for patient‐level ECE prediction. Unfold AI achieved 68% sensitivity, 78% specificity, 71% positive predictive value, and 75% negative predictive value. At the quadrant level, Unfold AI exceeded the AUC of MRI Likert scores for posterior (0.89 versus 0.82, *p* = 0.003), anterior (0.84 versus 0.80, *p* = 0.34), and all quadrants (0.89 versus 0.82, *p* = 0.002). The false negative rate of Unfold AI was lower than MRI in both the anterior (−60%) and posterior prostate (−40%).

**Conclusions:**

Unfold AI accurately predicted ECE risk, outperforming conventional methodologies. It notably improved ECE prediction over MRI in posterior quadrants, with the potential to inform nerve‐spare technique and prevent positive margins. By enhancing PCa staging and risk stratification, AI‐based cancer mapping may lead to better oncological and functional outcomes for patients.

## 1 INTRODUCTION

For men in the United States, prostate cancer (PCa) is the most frequently diagnosed cancer and the second‐most common cause of cancer death.^1^ Treatment paradigms have evolved in recent decades, but radical prostatectomy (RP) remains the most common therapeutic modality for intermediate and high‐risk PCa.^2^, ^3^ Extracapsular extension (ECE) is an important consideration during RP planning since it is associated with an elevated risk of cancer recurrence and adverse outcomes.^4^ In particular, ECE status often determines surgical margins and whether to spare the neurovascular bundles.^5^ Appropriately executed nerve sparing improves urinary and sexual function without diminishing cancer control.^6^, ^7^ Thus, accurate ECE identification is crucial to assure oncological efficacy and functional outcomes for RP patients.

Several predictive tools, such as preoperative MRI,^8^, ^9^, ^10^ PSMA‐PET/CT imaging,^11^, ^12^, ^13^ and various nomograms^14^, ^15^, ^16^, ^17^ estimate ECE risk. However, current paradigms are imperfect predictors of ECE, frequently over‐ or underestimating true PCa extent.^18^, ^19^, ^20^, ^21^ Furthermore, image‐based ECE prediction is subjective and dependent upon reader experience. To overcome these shortcomings, several artificial intelligence (AI)‐based tools for ECE detection have been developed in recent years.^22^, ^23^, ^24^ AI analysis is a promising alternative to current practice, and some initial success has been reported. However, extant models are not commercially available and rely on a single modality of data (MRI or clinical). AI that combines multi‐modal data and is readily available to clinicians has the potential to improve ECE risk assessment and impact patient outcomes.

Readily available software cleared by the United States Food and Drug Administration (Unfold AI, K221624, Avenda Health, Culver City, CA) uses an AI algorithm to visualize cancer probability in 3D. The Unfold AI model was trained to generate 3D cancer estimation maps (CEMs) using multi‐institutional, multi‐modal input data consisting of T2‐weighted MRI, prostate and MRI region of interest (ROI) segmentations, 3D biopsy locations, International Society of Urological Pathology Grade Group (GG), and serum prostate‐specific antigen (PSA). Additional information regarding the AI algorithm development, training, and validation are presented in Priester et al.^25^ In prior studies, Unfold AI was shown to improve intraprostatic PCa contours.^25^, ^26^ We hypothesized that the AI output could be used to predict ECE occurrence by assessing cancer probability adjacent to the prostate capsule.

## 2 MATERIALS AND METHODS

We conducted a retrospective single‐centre assessment of ECE detection with Unfold AI. We compared AI with conventional methodologies: MRI ECE assessment (1–5 Likert Score),^27^, ^28^, ^29^ ROI contact length, Partin Tables,^17^ the PRedicting ExtraCapsular Extension (PRECE) nomogram,^16^ and ^68^Ga‐PSMA‐11 PET/CT primary tumour stage (T stage). The ground truth presence of ECE in each quadrant was determined by pathologist review of whole‐mount histopathology.

### 2.1 Dataset Description

**FIGURE 1:**
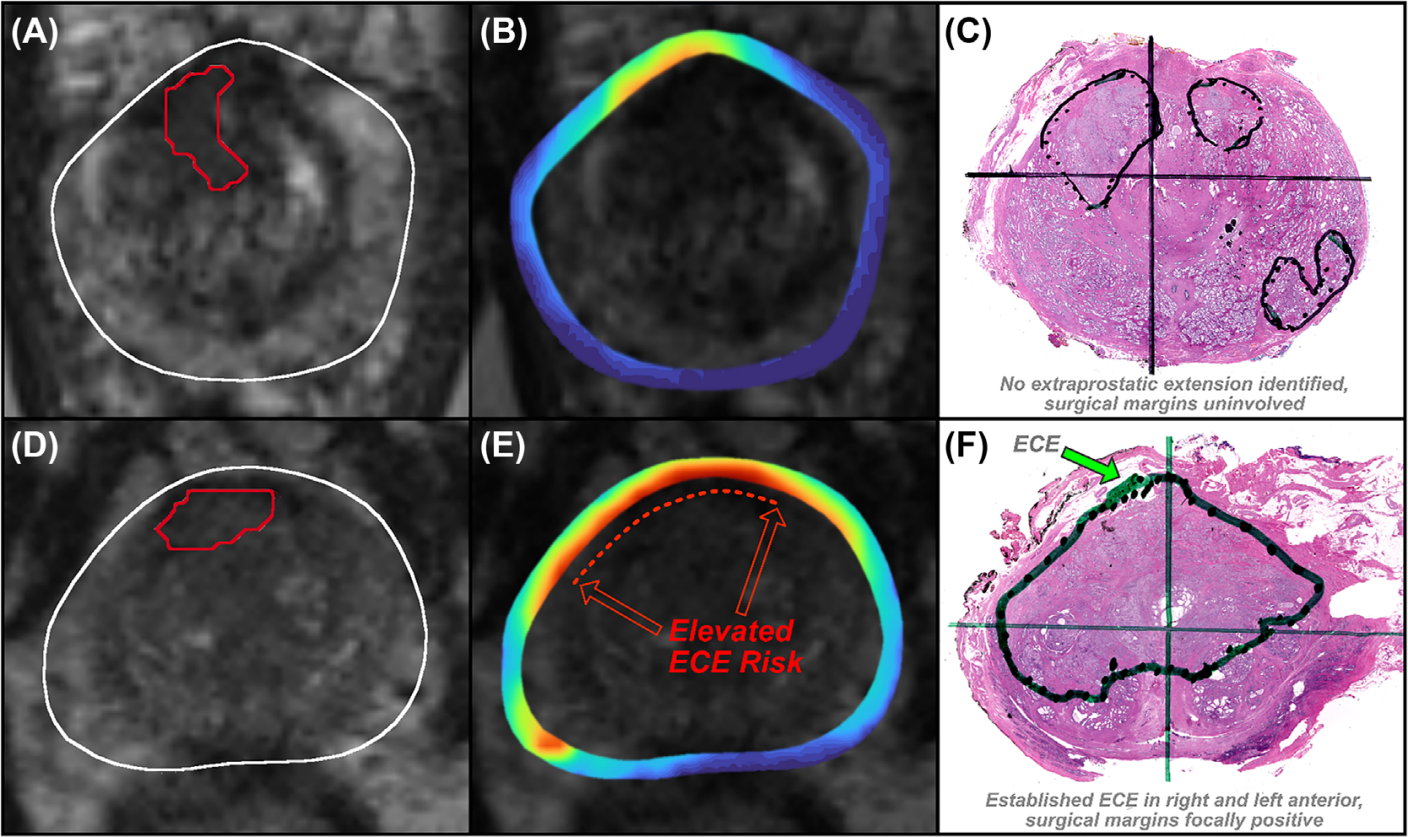
Two exemplary cases with similar MRI regions of interest, showing (A, D) T2‐weighted MRI, (B, E) Unfold AI ECE risk assessment, and (C, F) whole‐mount histopathology. The first case (A‐C) had low ECE risk on Unfold AI, no ECE on histopathology, and negative surgical margins. The second case had high ECE risk on Unfold AI, ECE on histopathology, and focally positive surgical margins. It is plausible that Unfold AI could have helped prevent positive margins for the second case.

In an IRB‐approved study, 241 patients consecutively accrued at the University of California, Los Angeles (UCLA) were retrospectively assessed. A radiologist prospectively interpreted multiparametric MRI obtained at either 1.5 (4% of cases) or 3 Tesla (96% of cases). The radiologist defined ROIs suspicious for PCa and assessed ECE risk via a 1–5 Likert scale. PSMA PET imaging was also performed in a subset of patients. All patients received preoperative biopsy via an MRI‐ultrasound fusion device between September 2016 and May 2022. Cores were sampled from ROIs and systematically as previously described.^30^ RP was then performed within one year of fusion biopsy. A genitourinary pathologist examined whole mount histopathology slides of the excised specimen to determine ground truth ECE status and location (Figure 1C, F).

The following inclusion criteria were applied to ensure data quality, a clinically relevant patient population, and compatibility with Unfold AI:

1. The patient received no prior surgical, ablative, or radiation treatment for PCa.
2. GG ≥ 2 PCa was detected on biopsy.
3. At least six biopsy cores were tracked and recorded during fusion biopsy, including ≥3 systematic cores and ≥1 targeted core.
4. Biopsy data was free from severe tracking, segmentation, and software errors.

**FIGURE 2:**
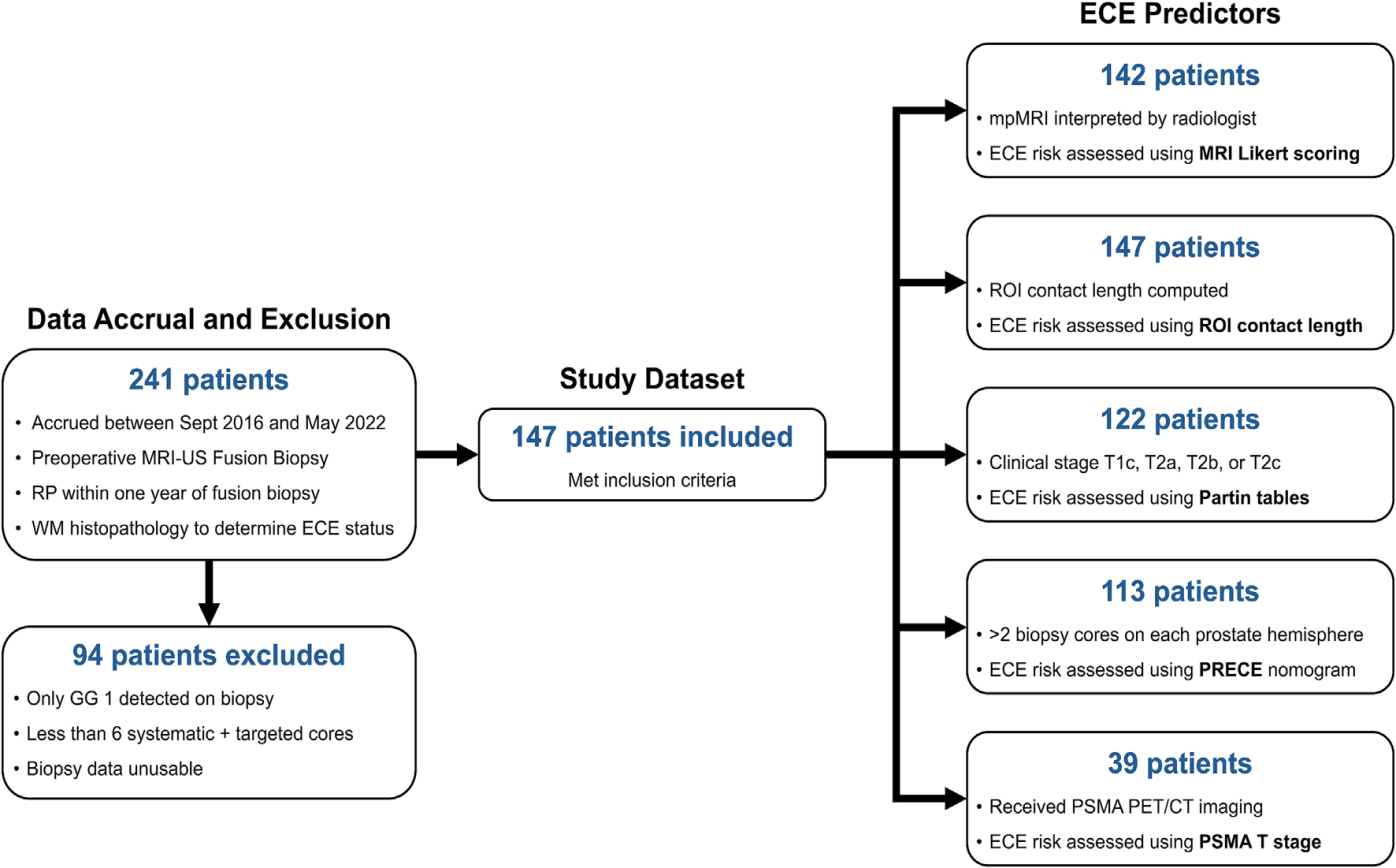
Flowchart illustrating dataset selection for this study. The final column describes the conventional ECE predictors and the number of patients available for comparison with Unfold AI.

One hundred and forty‐seven cases met inclusion criteria; the dataset selection process is illustrated in Figure 2 and population characteristics are summarized in Table 1.

**TABLE 1:** Patient characteristics (N = 147).

Characteristic		Data
**Years of Age**	Median (IQR)	71 (66–74)
**PSA (ng/ml)**	Median (IQR)	7.2 (5.2–11.0)
	<10	100 (68%)
	10–20	42 (29%)
	>20	5 (3%)
**Biopsy Cores Sampled**	Targeted (Median, IQR)	6 (4–7)
	Systematic (Median, IQR)	10 (7–11)
	Total (Median, IQR)	16 (14–17)
**Biopsy Grade Group (GG)**	GG 2	55 (37%)
	GG 3	47 (32%)
	GG 4	23 (16%)
	GG 5	22 (15%)
**Clinical T Stage**	<T2	123 (84%)
	≥ T2 and <T3	24 (16%)
**PI‐RADS Score** ^31^	3	17 (12%)
	4	40 (29%)
	5	83 (59%)
**MRI Likert Score (for ECE risk)**	1	10 (7%)
	2	55 (39%)
	3	35 (25%)
	4	24 (17%)
	5	18 (13%)
**PSMA T Stage**	T2	32 (82%)
	≥T3	7 (18%)

### 2.2 ECE Prediction Using Unfold AI

The Unfold AI algorithm incorporates multi‐modal input data: T2‐weighted MRI, PSA, 3D biopsy locations, and biopsy core pathology (GG, core length, and cancer length). Additional details on algorithm development, parameters, and validation have been previously reported.^25^ The AI software generates a 3D cancer estimation map, representing the probability of csPCa (defined as GG ≥ 2) in each prostate voxel. The CEM for each patient was downloaded and analysed using custom scripts written in Python.

The capsular contact length of MRI‐visible lesions has been previously reported to correlate strongly with ECE occurrence.^32^ Since Unfold AI is a more accurate reflection of tumour extent than MRI,^25^, ^26^ we hypothesized that ECE risk could be predicted using the CEM values of voxels intersecting the prostate capsule. Patient‐level ECE risk was estimated as the total csPCa probability of capsular voxels (Figure 1B, E). Similarly, quadrant‐level ECE risk was estimated as the total csPCa probability of capsular voxels in the left anterior, right anterior, left posterior, and right posterior quadrants.

### 2.3 ECE Prediction Using Conventional Methodologies

ECE risk was also assessed using conventional methods as detailed below.

1. MRI Likert score was derived from an expert radiologist's interpretation of multiparametric MRI. They assigned each case a Likert score between 1 and 5 according to the criteria in Table 2. This approach was used in lieu of PI‐RADS scores, which are optimized for tumour diagnosis rather than ECE detection and have been previously shown to over‐stage ECE.^33^
2. ROI contact length was defined as the maximum contact distance between the ROI(s) and the prostate. A point on the ROI was considered to be in contact if it lay within 1.5 mm of the prostate capsule.
3. ^68^Ga‐PSMA‐11 PET/CT imaging was acquired on a subset of patients. The apparent T stage was assessed jointly by an expert radiologist and nuclear medicine physician based on the distribution and relative uptake of PSMA.
4. Partin Table values were computed using clinical variables (GG, PSA, and clinical stage) as described by Tosoian et al.^34^ Partin table predictions were applicable for cases with clinical stages T1c‐T2c.
5. The PRECE nomogram by Patel et al.^16^ was used to estimate ECE risk using published logistic model coefficients for age, PSA, clinical stage, rate of PCa‐positive cores, rate of csPCa‐positive cores, rate of cores >60% cancer‐positive, and average percentage of cancer. The PRECE nomogram was only applicable for cases with a predetermined clinical stage and more than two biopsy cores in both the left and right prostate lobes.

**TABLE 2:** Likert Scoring of ECE risk for PI‐RADS ROIs.

Likert score	Criteria
1	ROI does not abut the capsule
2	ROI abuts or may abut the capsule
3	ROI has a broad base of capsular contact or bulges the capsule
4	ROI capsular contact is irregular or blurred
5	ROI has clear extraprostatic extension (gross or minimal)

In addition, quadrant‐level ECE was predicted on MRI using Likert Score. Each quadrant was assigned the highest Likert score among ROIs inside it (defined as intersecting ≥10% of the ROI volume). If a quadrant contained no ROI, a score of 0 was assigned.

### 2.4 Assessment of ECE Prediction Accuracy

The area under the curve (AUC) of the receiver operating characteristic (ROC) was used as the primary metric to assess ECE predictions. Following the application of a decision threshold closest to the (0,1) point on the ROC curve,^35^ secondary metrics were also calculated for each predictor: sensitivity, specificity, balanced accuracy, positive predictive value (PPV), and negative predictive value (NPV). All metrics were computed at both the patient and quadrant level for Unfold AI and the conventional comparator(s). Statistical significance was assessed using DeLong's test^36^ in Stata: Release 15 (College Station, TX: StataCorp LLC.), which compared the AUC of Unfold AI with conventional methods. Since conventional predictors were not available for every case (see Figure 2), each statistical test was performed individually on the sub‐population of cases with each predictor available.

## 3 RESULTS

### 3.1 Patient‐Level ECE Prediction

**FIGURE 3:**
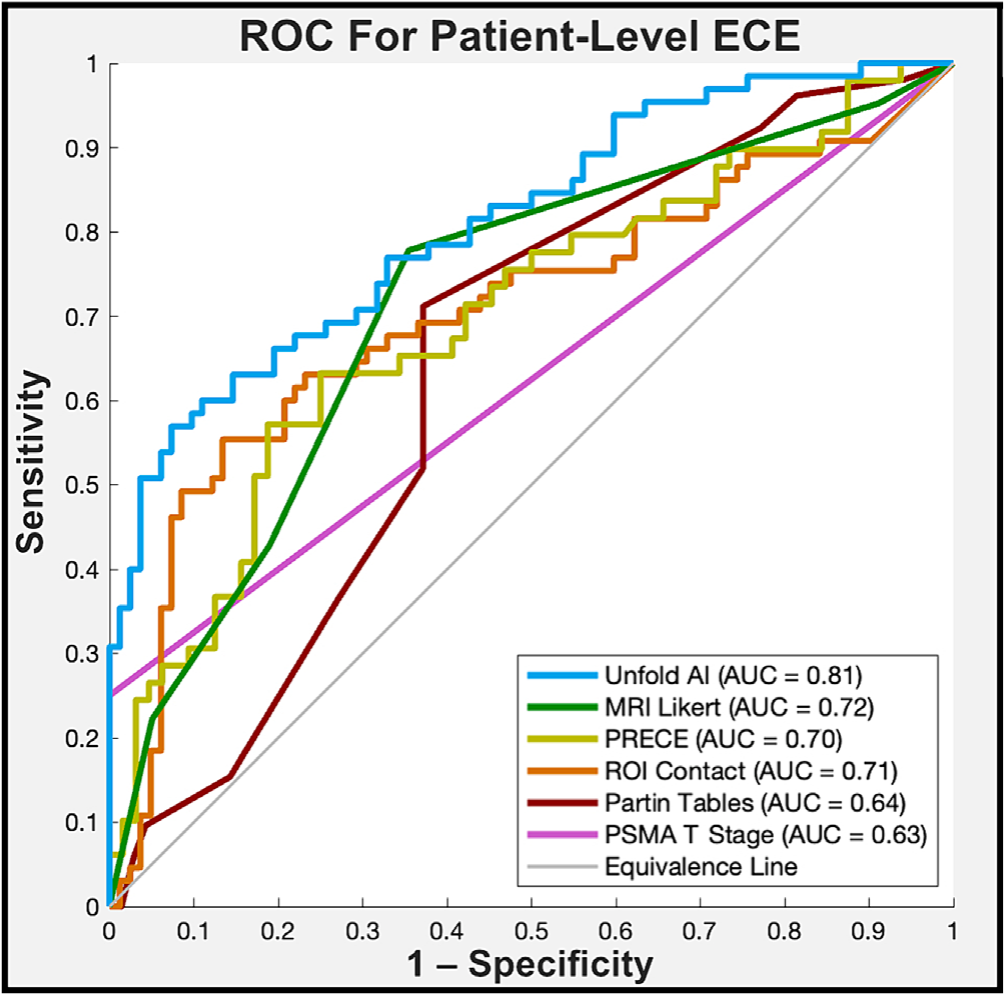
Receiver operating characteristic curves for patient‐level ECE prediction, computed using the data subset available for each metric (sample sizes vary; see Table 3).

Unfold AI had superior AUC to all other predictors (*p* < 0.05), outperforming them by 0.13 on average. Table 3 shows the AUC values for Unfold versus each conventional predictor, and Figure 3 shows the patient‐level ROC curves for all ECE predictors. When thresholded to a binary prediction of ECE, Unfold AI had an average sensitivity of 68%, specificity of 76%, balanced accuracy of 72%, PPV of 73%, and NPV of 70%. Secondary metrics for conventional comparators are listed in the Appendix A (Table A1).

**TABLE 3:** AUC measures and comparisons for patient‐level ECE prediction.

Predictor	Sample size	ECE prevalence	AUC		Std. Err.		*p*‐Value
			*Predictor*	*AI (same patients)*	*Predictor*	*AI (same patients)*	
Unfold AI	147	65 (44%)	‐	0.812	‐	0.036	‐
MRI Likert	142	63 (44%)	0.719	0.810	0.042	0.037	0.044
ROI Contact	147	65 (44%)	0.706	0.812	0.046	0.036	0.011
Partin Tables	122	52 (42%)	0.640	0.797	0.049	0.041	0.008
PRECE	113	49 (43%)	0.699	0.795	0.051	0.043	0.046
PSMA T Stage	39	28 (71%)	0.625	0.825	0.042	0.067	0.003

### 3.2 Quadrant‐Level ECE prediction

**FIGURE 4:**
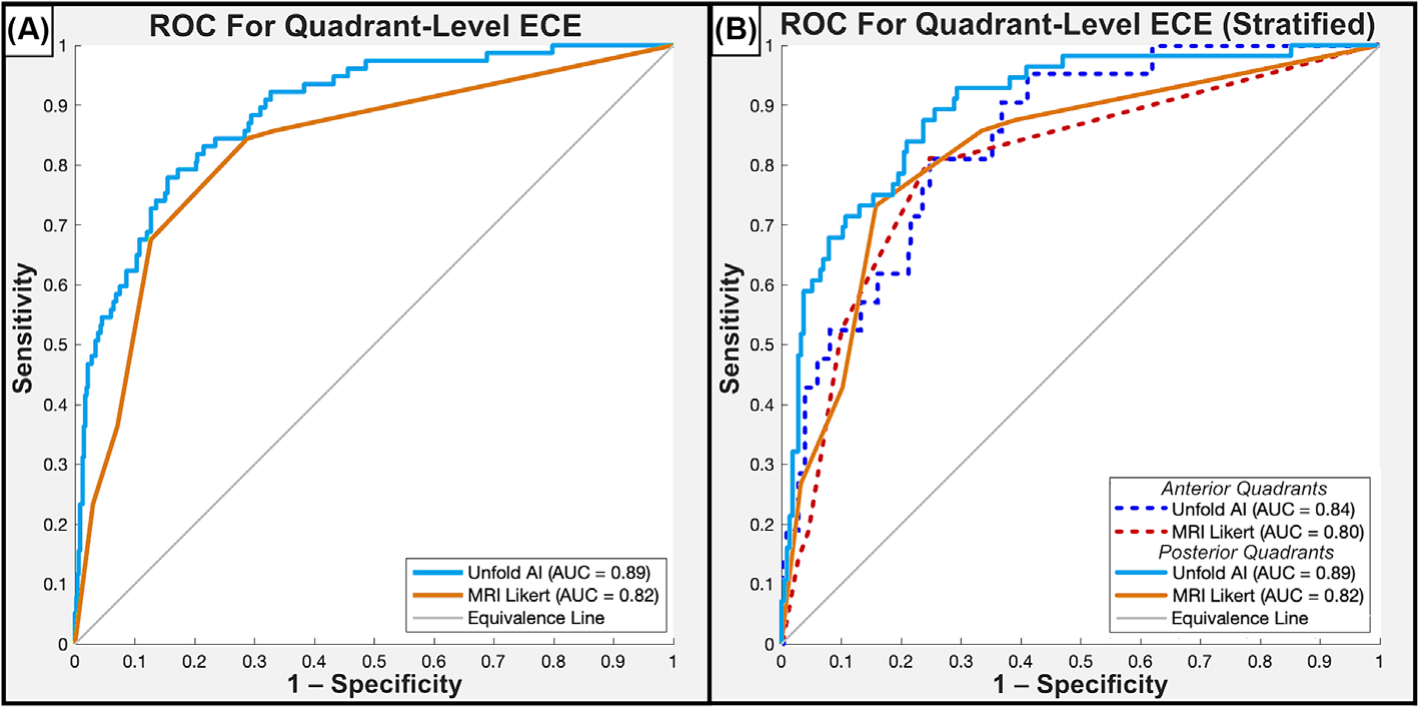
Receiver operating characteristic curves for quadrant‐level ECE prediction, analysing (A) all quadrants in aggregate, N = 542, and (B) quadrants stratified into anterior and posterior subgroups, N = 271.

**FIGURE 5:**
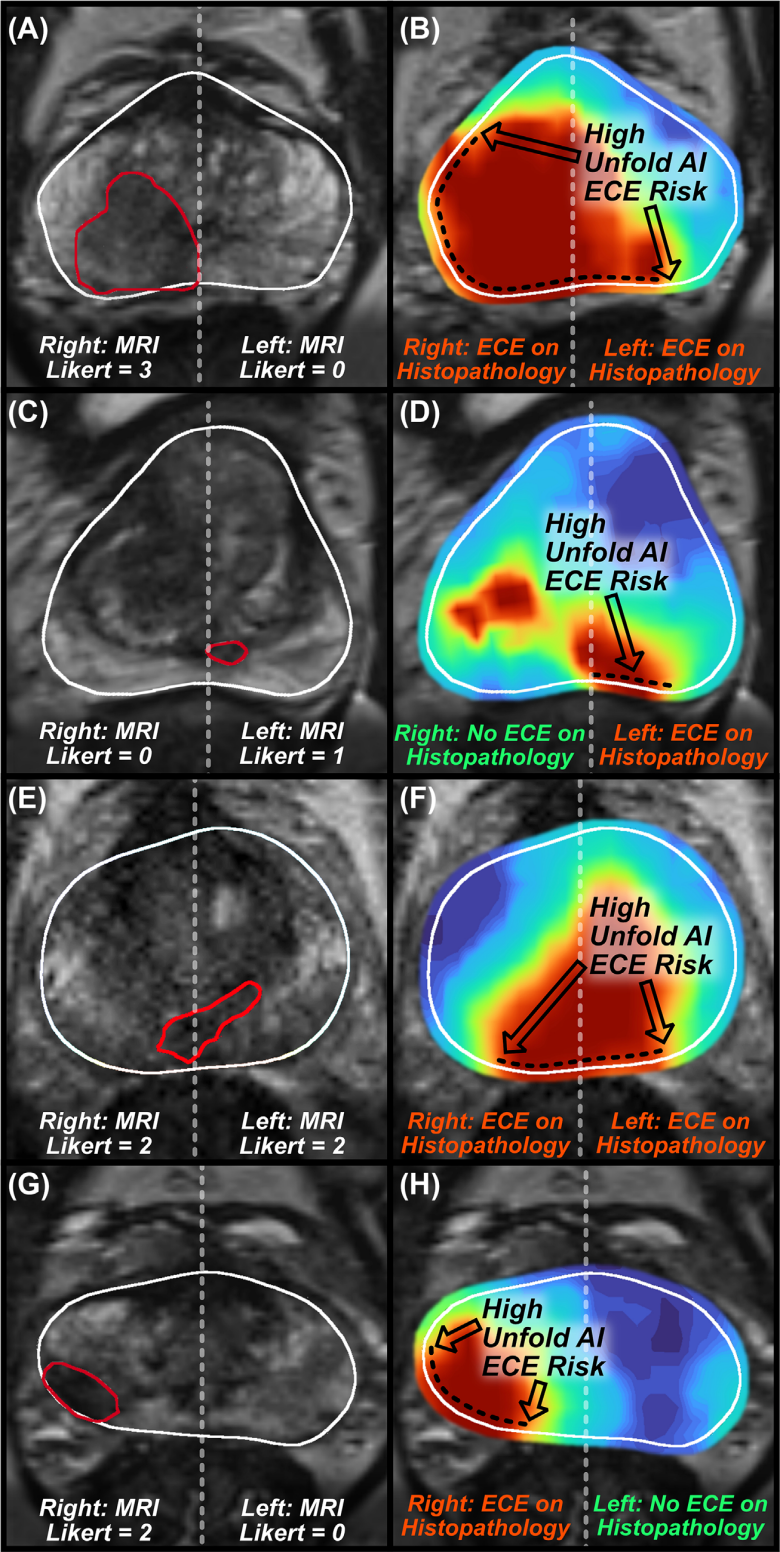
Example cases for which MRI failed (false negative) but Unfold AI succeeded (true positive) to predict posterior‐quadrant ECE. The left column shows MR images and MRI Likert Scores for the posterior quadrants, with the prostate outlined in white, the ROI outlined in red, and prostate midline annotated with a grey dotted line. The right column shows Unfold AI cancer estimation maps and histopathology ECE ground truth for posterior quadrants, and the region of highest ECE risk annotated with a black dotted line. Example cases include (A‐B) a case where MRI predicted ECE (Likert = 3) only in the right posterior, but Unfold AI successfully predicted bilateral ECE; (C‐D) a case where MRI predicted no ECE (Likert = 1), but Unfold AI successfully predicted ECE in the left posterior; (E‐F) a case where MRI predicted no ECE (Likert = 2), but Unfold AI successfully predicted bilateral ECE; and (G‐H) a case where MRI predicted no ECE (Likert = 2), but Unfold AI successfully predicted ECE in the right posterior.

The prevalence of ECE in the anterior and posterior prostate was 8% (21/271) and 21% (56/271) respectively. Unfold AI had superior AUC to MRI for ECE assessment in all quadrants (0.89 versus 0.82, *p* = 0.003) and posterior quadrants (0.89 versus 0.82, *p* = 0.002). The AUC of Unfold AI was also higher than MRI for anterior quadrants, though the difference did not achieve statistical significance (0.84 versus 0.80, *p* = 0.34). Figure 4 shows the ROC plots for quadrant‐based analysis. When thresholded to a binary prediction of ECE, Unfold AI achieved 84% sensitivity, 79% specificity, 82% balanced accuracy, 51% PPV, and 95% NPV for ECE prediction in posterior quadrants. Furthermore, in posterior quadrants, the false negative rate was 27% for MRI Likert scores and 16% for Unfold AI, a pronounced difference with important ramifications for RP planning. Example cases of false negative MRI findings but true positive Unfold AI findings are shown in Figure 5. Additional metrics are summarized in Table 4.

**TABLE 4:** Results for quadrant‐level ECE prediction.

Quadrants	Metric	Unfold AI	MRI Likert Score
**All quadrants** N = 542 ECE Prevalence = 77 (14%)	AUC (Std. Err.)	0.888 (0.020)	0.825 (0.026)
	Sensitivity	77.9%	67.5%
	Specificity	84.5%	87.3%
	Balanced Accuracy	81.2%	77.4%
	PPV	45.5%	46.8%
	NPV	95.9%	94.2%
**Anterior quadrants** N = 271 ECE Prevalence = 21 (8%)	AUC (Std. Err.)	0.843 (0.039)	0.800 (0.051)
	Sensitivity	81.0%	52.4%
	Specificity	75.2%	90.0%
	Balanced Accuracy	78.1%	71.2%
	PPV	21.5%	30.6%
	NPV	97.9%	95.7%
**Posterior quadrants** N = 271 ECE Prevalence = 56 (21%)	AUC (Std. Err.)	0.894 (0.023)	0.821 (0.032)
	Sensitivity	83.9%	73.2%
	Specificity	79.1%	84.2%
	Balanced Accuracy	81.5%	78.7%
	PPV	51.1%	54.7%
	NPV	95.0%	92.3%

## 4 DISCUSSION

In this study, we demonstrate that Unfold AI significantly outperforms MRI, ^68^Ga‐PSMA‐11 PET/CT, and nomogram‐based approaches to predicting and localizing ECE (Table 3). Though it is difficult to assess clinical impact in the absence of prospective data, Unfold AI has the potential to enhance decision‐making during radical prostatectomy. In particular, the ECE risk in each posterior quadrant may help determine whether the adjacent neurovascular bundles should be spared or resected. Compared with MRI, Unfold AI would have reduced the false negative rate from 27% to 16% in posterior quadrants, a 40% relative reduction (Table 4). Notably, MRI evaluation of ECE also performed well within this cohort. However, the performance of expert radiologists in an academic institution may not be broadly representative, and MRI sensitivity was much higher in the present study (0.73) than it has been reported historically (0.55).^37^ Despite recent evidence suggesting potentially improved ECE detection using PSMA vs MRI,^38^ ^68^Ga‐PSMA‐11 PET/CT underperformed in our study population. Though ^68^Ga‐PSMA‐11 PET/CT excels at the detection of metastases, it seems to struggle to distinguish organ‐confined from locally advanced disease. To date MRI remains the best available tool for ECE evaluation, despite a tendency to underestimate tumour size^20^ and insufficient resolution to identify microscopic ECE foci.^39^ There remains a pressing need to improve presurgical staging, a task AI is well suited to address.

Improving the assessment of ECE has immediate clinical implications. First, surgeons can utilize Unfold AI to implement nerve‐sparing to improve urinary and sexual outcomes^6^, ^7^ while maintaining oncologic outcomes. Second, ECE is a contraindication for focal therapy.^40^ Accurately determining ECE will appropriately select patients for surgery who may initially appear to be good focal therapy candidates on MRI (Figure 5G). Finally, Unfold AI provides a 3D map that can be displayed within the surgical robot during RP. This capability could improve clinical outcomes since similar maps based on MRI technology alone have enabled a reduction in surgical margins.^41^

The findings of this study are consistent with previous AI‐based ECE detection efforts, which reported AUC values of 0.72–0.88 and sensitivities of 76–82%.^24^, ^42^, ^43^, ^44^ Our approach compares favourably with prior work since it maps cancer risk in 3D, enabling localization and visualization of tumour stage and extent. Indeed, the performance of Unfold AI is remarkable considering that it was developed to map intraprostatic cancer risk, and never explicitly trained for ECE detection. Its success is likely attributable to its multi‐modal nature, wherein predictions are made using diverse minimally correlated data from imaging, biopsy, and biomarkers. The use of multi‐modal data may even reduce dependence on the quality of any one data source. Though nomograms such as PRECE attempt to similarly incorporate multi‐modal data, they do not fully leverage imaging and 3D information. Conversely, ^68^Ga‐PSMA‐11 PET/CT and MRI entail 3D imaging but lack multi‐modal data correlates and are susceptible to inter‐reader variability.

The promising performance of Unfold AI warrants future development efforts. The AI model could be enhanced through the incorporation of additional data sources such as diffusion‐weighted MRI, perfusion MRI, ultrasound images, and high‐resolution images of biopsy histopathology. Furthermore, Unfold AI and conventional predictors could be incorporated into a combined model, which may outperform Unfold AI alone. Lastly, this study demonstrates the potential of AI cancer mapping to improve upon multiple aspects of PCa management, with no need to develop task‐specific models. Future studies could investigate the use of Unfold AI cancer estimation maps to enhance therapy selection, radiation dosing, PCa staging, biopsy planning, and PCa progression prediction.

This retrospective study had several limitations worth noting. Firstly, though the AI model was trained on multi‐institutional data, all ECE cases were derived from a single institution (UCLA). Furthermore, the definition of ECE ground truth relied on the interpretation of a single experienced pathologist. Follow‐up multicentre studies, with diverse populations of both patients and physicians, are warranted. Secondly, our analysis did not distinguish between focal and established ECE, which have different ramifications for both treatment and prognosis.^45^ Future efforts should a entail also predicting ECE extent. Third, factors such as patient preference and surgeon experience can strongly influence nerve‐spare technique and positive margin rates. The true clinical impact of Unfold AI is impossible to predict with retrospective data, and thus a prospective study is currently being planned. Lastly, though Unfold AI outperformed MRI in posterior quadrants, differences in anterior quadrant predictions were not statistically significant. This may be explained by the scarcity of anterior quadrant ECE (only 8%) in the dataset, likely underpowering comparisons. Also, a key advantage of Unfold AI is the incorporation of tracked biopsy data, which is far more prevalent in the posterior than anterior gland. In the absence of thorough biopsy sampling, the relative benefit of Unfold AI in the anterior gland may be diminished.

## 5 CONCLUSIONS

Unfold AI shows promise as a means of assessing ECE risk, particularly in posterior quadrants. It significantly outperformed alternative approaches including MRI, ^68^Ga‐PSMA‐11 PET/CT, and other commonly used nomograms. Thus, Unfold AI has the potential to improve prostatectomy planning and inform nerve resection technique. By enhancing PCa staging and risk stratification, AI‐based cancer mapping could improve both oncological efficacy and quality of life for patients with prostate cancer.

## AUTHOR CONTRIBUTIONS

**Alan Priester:** Methodology; data collection; formal analysis and investigation; writing—original draft preparation; writing—review and editing. **Sakina Mohammed Mota:** Methodology; data collection; formal analysis and investigation; writing—original draft preparation; writing—review and editing. **Kyla P. Grunden:** Data collection. **Joshua Shubert:** Methodology; data collection; writing—review and editing; supervision. **Shannon Richardson:** Data collection. **Anthony Sisk:** Data collection. **Ely R. Felker:** Data collection. **James Sayre:** Formal analysis and investigation; writing—review and editing. **Leonard S. Marks:** Data collection; writing—review and editing. **Shyam Natarajan:** Methodology; data collection; writing—review and editing; supervision. **Wayne G. Brisbane:** Methodology; data collection; writing—original draft preparation; writing—review and editing; supervision.

## CONFLICT OF INTEREST STATEMENT

Unfold AI is commercially available cancer mapping software provided by Avenda Health. Dr. Brisbane receives no financial compensation from Avenda Health. He had complete control of the data and supervised manuscript preparation. Dr. Sayre performed all statistical analyses. Dr. Priester and Dr. Mota were responsible for deriving AI‐based ECE risk prediction.

Dr. Priester, Dr. Mota, and Mr. Shubert are employees at Avenda Health.

Dr. Natarajan and Dr. Marks are co‐founders of Avenda Health.

## Figures and Tables

**FIGURE B1:**
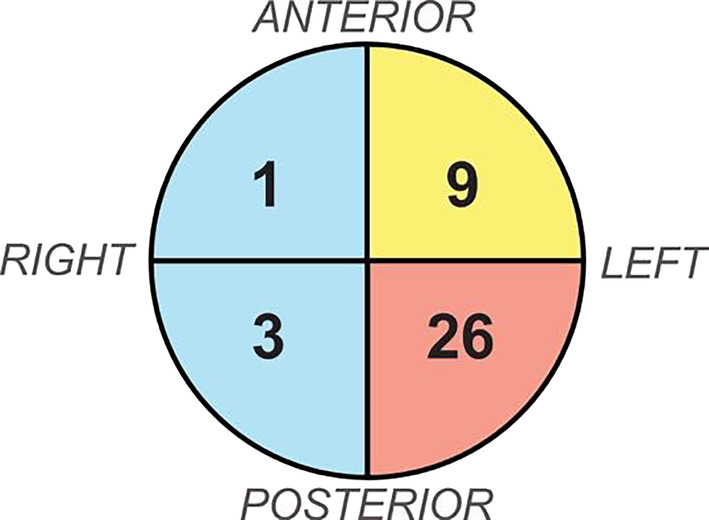
Unfold AI ECE risk metric values for an illustrative case, which can be used for prospective ECE risk assessment.

## Appendix

Appendix A Table A1 shows the performance of Unfold AI versus conventional comparators when thresholded using the ROC curve points closest to (0,1). Unfold AI exhibited higher sensitivity than ROI contact length, PRECE, and PSMA T stage and higher specificity than all except PSMA T stage. It also demonstrated a higher balanced accuracy of 6% on average than all conventional methods. Lastly, it had higher PPV than all predictors except PSMA T stage and higher NPV than all others except MRI Likert score.

**TABLE A1:** Secondary metrics and comparisons for patient‐level ECE prediction.

Predictor	ECE Prevalence	Sensitivity		Specificity		Balanced Accuracy		PPV		NPV	
		*Predictor* *(%)*	*AI* *(%)*	*Predictor* *(%)*	*AI* *(%)*	*Predictor* *(%)*	*AI* *(%)*	*Predictor* *(%)*	*AI* *(%)*	*Predictor* *(%)*	*AI* *(%)*
Unfold AI	65/147 (44%)	‐	67.7	‐	78.0	‐	72.9	‐	71.0	‐	75.3
MRI Likert	63/142 (44%)	77.8	68.3	64.6	77.2	71.2	72.7	63.6	70.5	78.5	75.3
ROI Contact	65/147 (44%)	63.1	67.7	76.8	78.0	70.0	72.9	68.3	71.0	72.4	75.3
Partin Tables	52/122 (42%)	71.2	63.5	62.9	80.0	67.0	71.7	58.7	70.2	74.6	74.7
PRECE	49/113 (43%)	63.3	65.3	75.0	79.7	69.1	72.5	66.0	71.1	72.7	75.0
PSMA T Stage	28/39 (71%)	25.0	75.0	100.0	63.6	62.5	69.3	100.0	84.0	34.4	50.0

## Appendix

Table B1 shows the ECE positive predictive value and 95% confidence intervals of the Unfold AI metric for anterior and posterior quadrants. The metric ranges represent low (1%), moderate (10%), and elevated (25%–50%, respectively) ECE risk. These ranges were selected to facilitate prospective use, enabling urologists to adjust their surgical technique according to the estimated ECE risk in each quadrant. The methodology of Clopper and Pearson^46^ was used to obtain 95% exact confidence limits for the PPV proportions.

**TABLE B1:** Quadrant‐level positive predictive value of the Unfold AI metric for ECE prediction.

	Unfold AI metric	<3.5	3.5–18	>18
	ECE risk	Low	Moderate	High
PPV *(Mean, 95% CI)*	Posterior*	1% *(0%*–*6%)*	10% *(4%*–*17%)*	50% *(40%*–*60%)*
	Anterior	1% *(0%*–*4%)*	10% *(5%*–*18%)*	25% *(13%*–*40%)*

* For simplicity of interpretation, posterior metric values were linearly transformed (y = 0.71x + 0.94) to match the anterior quadrant PPV at the “low” and “moderate” risk stratification boundaries. This transform is automatically applied for risk values displayed via Unfold AI.

Figure B1 shows quadrant‐level Unfold AI ECE risk metric values for an illustrative case. By cross‐referencing this case with Table B1, ECE risk would be prospectively assessed as follows:

- Left posterior (metric value = 26): high risk of ECE (mean 50%, CI 40%–60%)
- Left anterior (metric value = 9): moderate risk of ECE (mean 10%, CI 5%–18%)
- Right posterior (metric value = 3): low risk of ECE (mean 1%, CI 0%–6%)
- Right anterior (metric value = 1): low risk of ECE (mean 1%, CI 0%–4%)
